# Risk Factors Associated With Osteonecrosis of the Femoral Head in Patients With Sickle Cell Disease: A Systematic Review and Meta-Analysis

**DOI:** 10.7759/cureus.76734

**Published:** 2025-01-01

**Authors:** Mahmoud Mohammed Hassaan, Alhassan H Hobani, Hanan A AlKaabi, Ahmad A Shugairi, Khlood K Alattas, Mohsen J Zaylaee, Hanen I Alsuri, Mohammad S Alnejaidi, Raghad M Aljuaid, Hayam A Alzahrani, Fatema S Mohamed Mahfoodh, Muteb N Alotaibi, Yazeed M Aldalbahi, Abdulmalik M Almukhashi, Mohammad A Alhazmi

**Affiliations:** 1 Surgery, Jazan University, Jazan, SAU; 2 College of Medicine, Jazan University, Jazan, SAU; 3 College of Medicine, King Khalid University, Abha, SAU; 4 College of Medicine, King Abdulaziz University, Jeddah, SAU; 5 Collage of Medicine, Taif University, Taif, SAU; 6 College of Medicine, Batterjee Medical College, Jeddah, SAU; 7 College of Medicine, Alexandria University, Alexandria, EGY; 8 Collage of Medicine, Alfaisal University, Riyadh, SAU; 9 College of Medicine, Imam Muhammad Ibn Saud Islamic University, Riyadh, SAU

**Keywords:** aseptic necrosis, femoral head, osteonecrosis, predictor, risk factor, sickle cell disease

## Abstract

Osteonecrosis of the femoral head (ONFH) is a serious complication in patients with sickle cell disease (SCD), secondary to the peculiar pathophysiology of the disease. SCD is characterized by stiff and adhesive RBCs, disturbing blood circulation, and causing ischemia and microvascular damage. Understanding the key risk factors contributing to ONFH in this population is critical for developing targeted interventions to improve outcomes in this population. This study aimed to identify and analyze the key risk factors associated with osteonecrosis of the femoral head (ONFH) in patients with sickle cell disease (SCD) through a comprehensive systematic review and meta-analysis. This meta-analysis used the Preferred Reporting Items for Systematic Reviews and Meta-Analyses (PRISMA) tool to search and select the most relevant studies from electronic databases, like Web of Science, Google Scholar, PubMed, Cochrane Library, Embase, and MEDLINE, based on the study focus. The following medical phrases were used in the search: femoral head, aseptic necrosis, osteonecrosis, predictor, risk factor, and sickle cell disease. The study included six studies with a total of 581 participants. Four significant risk factors for ONFH in SCD were identified as follows: genotype (risk ratio: 1.09, 95% CI: 0.94-1.27), elevated hemoglobin levels (mean difference: 0.30, 95% CI: -0.03 to -0.63), hip pain (risk ratio: 1.11, 95% CI: 0.80-1.55), and acute chest pain (mean difference: 1.17, 95% CI: 0.87-1.47). Statistical analysis showed low heterogeneity across studies (I²: 0%, p < 0.05), indicating consistent findings. The ONFH group was more significantly affected by these factors than the comparator group. The meta-analysis revealed an intricate interplay of vascular, hematologic, and systemic factors contributing to ONFH of SCD with the four significant risk factors mainly genotype, elevated hemoglobin levels, hip pain, and acute chest pain. Therefore, it is important to carry out regular check-ups and focus on specific treatments to manage these clearly identified risk factors, which can help slow down the progression of the disease.

## Introduction and background

Osteonecrosis of the femoral head (ONFH) is a debilitating condition characterized by the death of bone tissue due to disrupted blood supply, leading to significant pain and functional limitations [1]. It is particularly prevalent in patients with sickle cell disease, a genetic disorder causing red blood cells to become stiff and adhesive, impairing circulation, and increasing the risk of ischemic complications [1]. Sickle cell disease increases the risk of ONFH due to impaired blood flow and repeated ischemic events, causing pain, reduced mobility, and a high likelihood of surgical interventions like hip replacement [2]. This disruption of circulation, particularly in the subchondral region, often leads to the suppression and eventual death of bone cells [1]. The disorder is typically associated with excessive intraosseous pressure, ischemia of the femoral head, and metabolic imbalances that disrupt the equilibrium between bone resorption and remodeling [2]. The most often performed surgical treatments include total hip replacement (THR), osteotomy, vascularized bone graft, tissue engineer material transplantation, and core decompression (CD). In SCD patients, ONFH can progress rapidly, leading to femoral head collapse and severe hip joint pain [3].

Osteonecrosis of the femoral head (ONFH) is a significant global health concern, particularly among high-risk populations such as individuals with sickle cell disease (SCD). Its socioeconomic impact is substantial, affecting productivity, quality of life, and healthcare costs, especially for younger SCD patients. Progressive ONFH is the leading cause of total hip replacement in this group [4]. Currently, there are no established medical interventions for ONFH in SCD patients [5]. Treatment options are generally limited to pain management with prescription medications, physical therapy, and early consideration of hip surgery [6]. However, a deeper comprehension of the risk factors for ONFH in SCD patients is required to improve outcomes through public awareness campaigns and preventative measures. Previous studies estimate that 10-30% of individuals with SCD worldwide are affected by ONFH, largely due to a combination of factors, such as bone marrow expansion, chronic hemolysis, microvascular occlusion, and corticosteroid use [7-9].

Necrosis, infarction, and infection are among the bone and joint issues that affect about 31% of SCD patients [10]. The most common issue linked to SCD is bone infection. Furthermore, a notable association has been observed between ONFH and a history of leg ulcers [11]. SCD patients and those with elevated hemoglobin levels are more vulnerable to the negative effects of ONFH [12]. When it comes to their hospital stays and pain episodes, SCD patients with avascular ONFH report longer stays than their non-ill peers [13]. Furthermore, compared to those without avascular necrosis, SCD patients with this consequence experience euglobulin clot lysis for a much longer period [14,15]. Considering the range of results found in various studies about the risk factors linked to femoral head osteonecrosis in SCD patients, it is critical to fully understand and address these variables to improve patient care and outcomes. The purpose of this meta-analysis was to identify and quantify key risk factors contributing to ONFH in patients with sickle cell disease. By synthesizing data from multiple studies, this research aimed to improve understanding and guide future interventions for this vulnerable population.

## Review

Materials and methods

Using the Preferred Reporting Items for Systematic Reviews and Meta-Analyses (PRISMA) tool, this systematic review and meta-analysis involved searching and selecting the most relevant studies from electronic databases, like Web of Science, Google Scholar, PubMed, Cochrane Library, Embase, and MEDLINE, based on the study focus. The medical phrases used in the search are as follows: femoral head, aseptic necrosis, osteonecrosis, predictor, risk factor, and sickle cell disease. The review examined English-language research published between January 2010 and the present, emphasizing finding risk factors for femoral head osteonecrosis in SCD patients.

Selection Criteria

Two seasoned researchers independently evaluated the articles’ full text, research titles, and abstracts for eligibility. Where there were disagreements, a third senior researcher was involved to reach an agreement.

The inclusion criteria involved comparing patients with osteonecrosis of the femur head and SCD with any other group with a single health issue, publications written in the English language that detail adverse events experienced by both the patients and comparators, randomized controlled trials, case-controlled studies, and clinical trials.

Case reports, case series, conference proceedings, non-English language publications, and studies with unavailable full-text articles were excluded.

Data Extraction Criteria

The Cochrane Consumers and Communication Review Group template was used to construct the data extraction sheet [16]. Two different investigators independently completed the matching sheet after obtaining the relevant data. Following that, discrepancies were resolved by agreeing, and a third researcher was asked to assess the accuracy of the combined data. Name of the lead author, year of publication, nation/region where the study was carried out, sample size, interventions, inclusions, and results were among the extracted data. When additional details about the studies were needed, the reviewers directly contacted the authors to obtain further information. Their collaboration made this research to be successful.

Statistical Analysis

The data's degree of diversity was shown by the I^2^ value in the analysis, which was carried out with the Review Manager program version 5.3.1 (Copenhagen, Denmark: The Nordic Cochrane Centre). A high I^2^ (>50%) and a significant level of p < 0.05 suggested substantial heterogeneity among the studies [17]. Fixed effect models were used when the I^2^ was more than 50%; otherwise, random effect models were employed. In trials where outcome event data were not available, treatment findings were translated into mean differences (MDs) or standardized mean differences (SMDs) and 95% confidence intervals [18]. While publication bias was assessed using a funnel plot, the risk of bias in research was visually represented by a bar graph [19]. The investigation of heterogeneity in subgroup results was also conducted using forest plots.

Results

**Figure 1:**
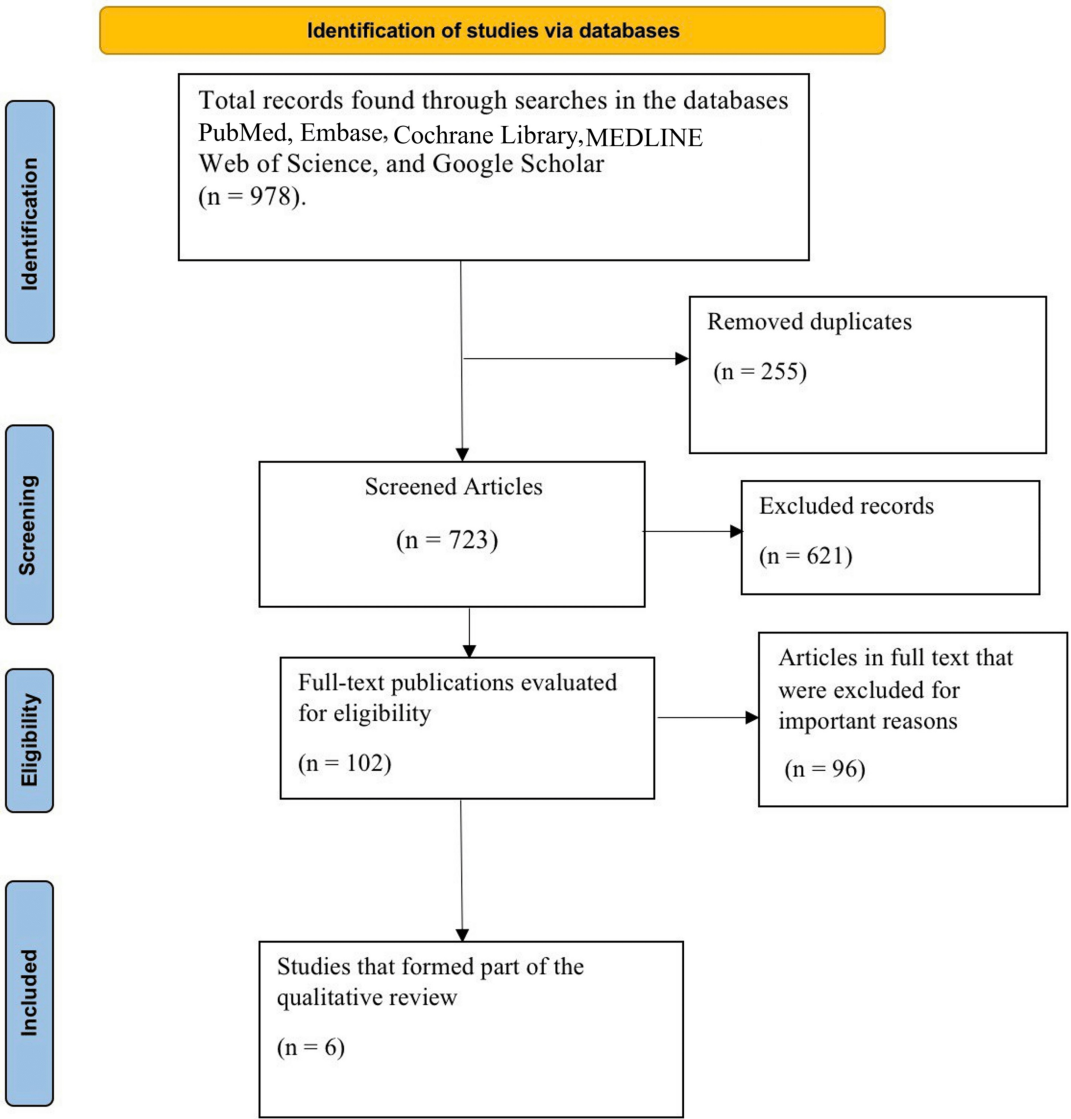
Preferred Reporting Items for Systematic Reviews and Meta-Analyses (PRISMA) flow diagram.

Initially, the search across several databases yielded 978 studies in total. Overall, 255 duplicate articles were excluded, and then after titles and abstracts screening, 621 papers were excluded due to noncompliance with the inclusion criteria. The full text of 102 papers was then examined to determine their eligibility. Further, 96 of 102 research papers were excluded for different reasons, such as studies not published in the English language, studies with no full-text availability, case reports, and conference abstracts. In the end, this meta-analysis included six studies. A flow diagram shows the entire process of selection (Figure 1).

Study Characteristics

The features of the studies that were examined to determine the risk factors for femoral head osteonecrosis in sickle cell disease patients are shown in Table 1. The six research papers included in the analysis were conducted globally and published in the English language. Overall, 581 participants were involved in the six studies. The columns of Table 1 provide an overview of the overall features of the research projects, while each row focuses on the distinctive features of the individual studies.

**Table 1:** Characteristics of the included studies. SCD: sickle cell disease; PJI: prosthetic joint infection; AVN: avascular necrosis; HB: hemoglobin; VOEs: vaso-occlusive pain events; TPO: thyroid peroxidase; ONFH: osteonecrosis of the femoral head; ATMP: advanced therapeutic products; THR: total hip replacement; HHS: Harris Hip Score

Study	Region	Sample size	Study design	Intervention	Inclusion	Outcome/results
Yu et al. (2016) [20]	United States	174	Retrospectively study	Computed tomography scanning	Individuals with SCD who exhibit symptoms of AVN and those who did not exhibit symptoms of AVN.	Increased urgent care visits and admissions were caused by acute venous nephropathy, which was linked to high utilization history, acute chest syndrome, pneumonia, hydroxyurea medication, and long-term transfusion.
Mallet et al. (2018) [21]	France	25	Retrospective study	Surgical treatment	Patients are required to be at skeletal maturity (closed triradiate cartilage on radiographs) at the most recent examination and have a minimum two-year follow-up to be eligible.	Both procedures resulted in good functional outcomes, with a mean HHS of 81 (SD 17) and 75% of hips being congruent at skeletal maturity.
Narayana et al. (2023) [22]	India	74	Retrospective study	Surgical correction	Acute venous nephropathy was established by imaging records in patients with severe chronic obstructive pulmonary disease (SCD) and chronic joint pain (femoral or humeral head).	Symptomatic AVN was identified as a risk factor for acute care utilization in SCD patients. More research is required to ascertain whether avascular necrosis prevention and early treatment measures can enhance outcomes for people with SCD, given that it is a potentially modifiable component.
Farook et al. (2019) [23]	United Kingdom	34	Case-controlled study	Surgery	A senior author and consultant hematologist assessed patients at a combined sickle cell clinic before surgery, six weeks after surgery, and once a year.	Revision surgery was performed on 16 patients (17.6%), of whom two (5.8%) had prosthetic joint infections and four (11.7%) had acetabular osteolysis.
Gómez-Barrena et al. (2021) [24]	France, Germany, Italy, and Spain	42	Multicenter clinical trial	Surgical treatment	The study included patients with femoral head osteonecrosis who were less than six months old, between the ages of 18 and 65 years, completed an informed consent form, were aware of the study's limitations, and had health insurance.	There were no serious side effects associated with ATMP, and at 12 months, 16 of 20 individuals still had spherical heads and demonstrated bone repair. Total hip replacement was necessary for three hips, and at five years, 16 of 21 showed no THR or development.
Tanabe et al. (2019) [25]	Atlanta	232	Case-controlled trial	Surgery	Hospitalization and ED use among patients aged 18 years and older.	In patients with SCD, the presence of VOEs in the bone's microcirculation was found to have caused thrombosis, infarction, and ultimately necrosis.

Quality of the Items of the Assessed Studies

**Figure 2:**
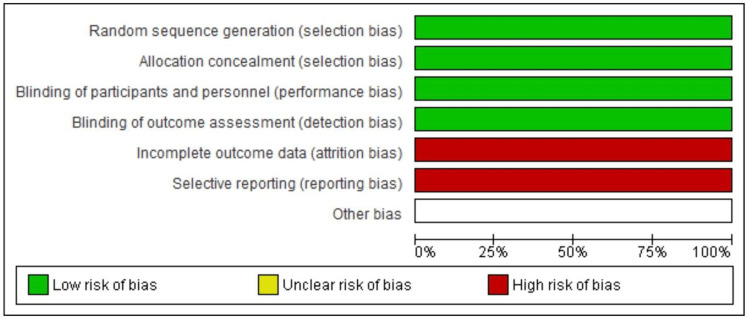
Risk of publication bias of the studies item.

Figure 2 presents a bar graph summarizing the quality of items used in two clinical trial studies included in this research. Cochrane risk of bias assessment tool for randomized controlled trials (RCTs) was used in the evaluation. Green bars denote a low risk of bias, red bars denote a high risk of bias, and white bars, symbolized by a yellow square box, indicate an unclear risk of bias. Specifically, four of the seven items used in the two studies indicate a minimal risk of bias. However, one of the seven items had an unclear risk of bias, while two of the seven items of the assessed study had a high risk of bias [24]. The Newcastle-Ottawa Scale (NOS) was employed as a standardized quality assessment tool, following the guidelines described by Wells et al. [26]. This scale allows for objective evaluation of cohort studies, with a specific focus on selection, comparability, and outcome assessment. On the basis of this, four of the seven items assessed in the included studies met good quality standards, suggesting that the overall quality of the included studies was generally good.

The quality of the cohort, case-controlled, prospective, and retrospective studies was assessed using the Newcastle-Ottawa Scale (NOS) (Table 2). According to the findings, the majority of the studies (five of nine) had a low risk of bias [20,21,23]. However, three of the nine studies showed a moderate risk of bias [22,25]. Cut-off points for study quality were based on NOS guidelines, which categorize studies with scores of 7-9 as low risk, ensuring robust data reliability (Table 2).

**Table 2:** The Newcastle-Ottawa Scale for the included cohort prospective and retrospective studies. Q1: Representativeness of the exposure cohort. Q2: Selection of the non-exposure cohort. Q3: Ascertainment of exposure. Q4: Demonstration that outcome of interest was not present at the start of the study. Q5: Comparability of the cohort based on the design or analysis. Q6: Assessment of outcome. Q7: Was follow-up long enough for outcomes to occur? Q8: Adequacy of follow-up of cohorts. Each correct item of the selection and outcome was awarded a single asterisk (*), while comparability was awarded up to two asterisks (**). Dash (-) indicates missing items.

Study	Section				Comparability	Outcome			Quality score	Risk of bias (0-3, high; 4-6, moderate; 7-8, low)
	Q1	Q2	Q3	Q4	Q5	Q6	Q7	Q8		
Yu et al. (2016) [20]	*	-	*	*	**	*	*	*	7	Low risk
Mallet et al. (2018) [21]	*	*	*	*	*	*	*	*	7	Low risk
Narayana et al. (2023) [22]	*	*	*	-	*	*	-	*	6	Moderate risk
Farook et al. (2019) [23]	*	-	*	*	**	*	*	*	8	Low risk
Tanabe et al. (2019) [25]	*	-	*	-	*	*	*	*	8	Moderate risk

Funnel Plots

**Figure 3:**
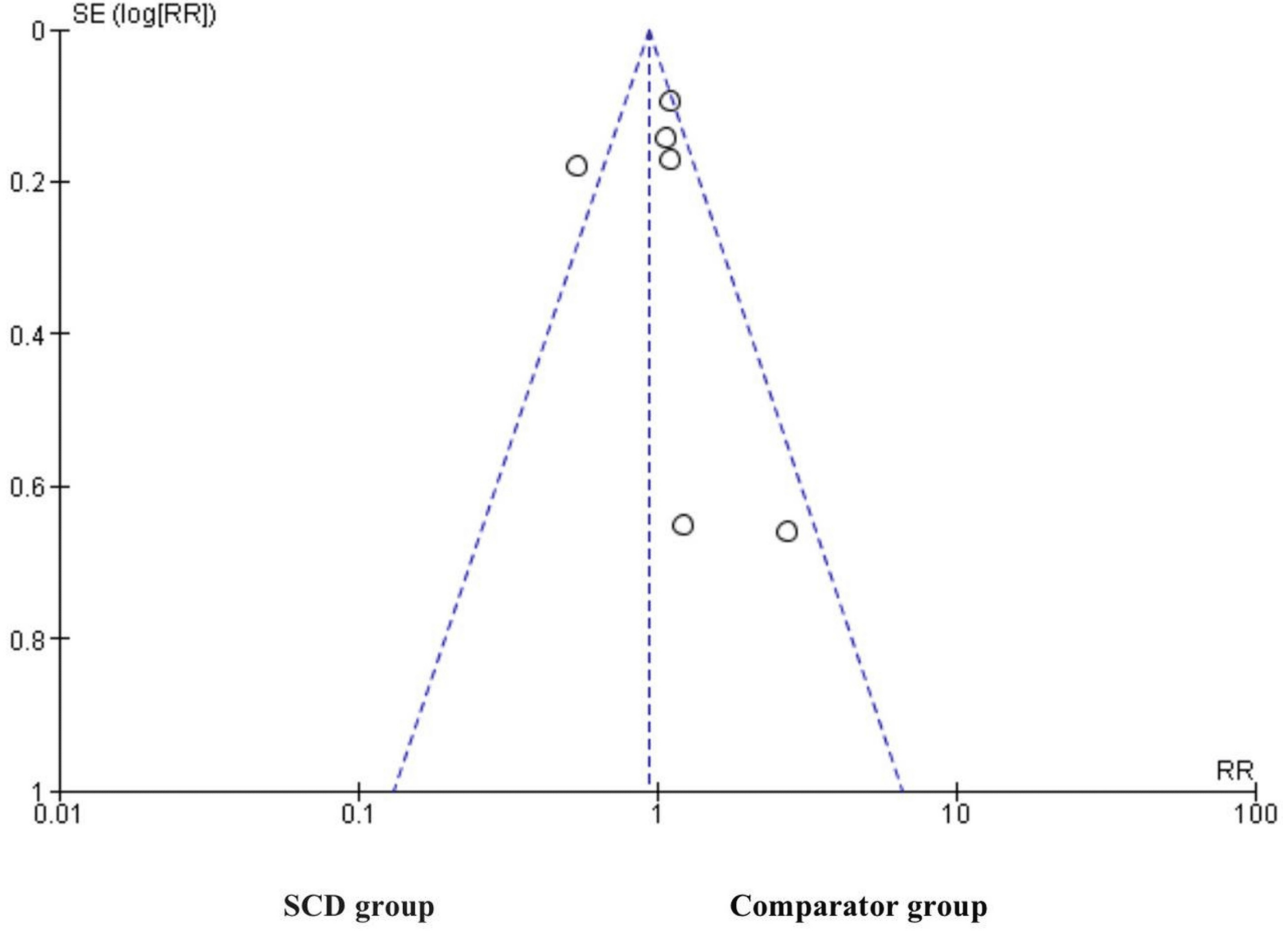
Funnel plot displaying publication bias. The X-axis represents the standard error, and the Y-axis represents the effect size. SCD: sickle cell disease; SE: standard error; RR: relative risk

Figure 3 shows a symmetrical funnel plot with most of the studies aligned to the right compared to the left. This implies that the effect size of the included studies was more on the right side. This suggests that there is a chance of publication bias in favor of the comparator group.

Meta-Analysis Outcome

**Figure 4:**
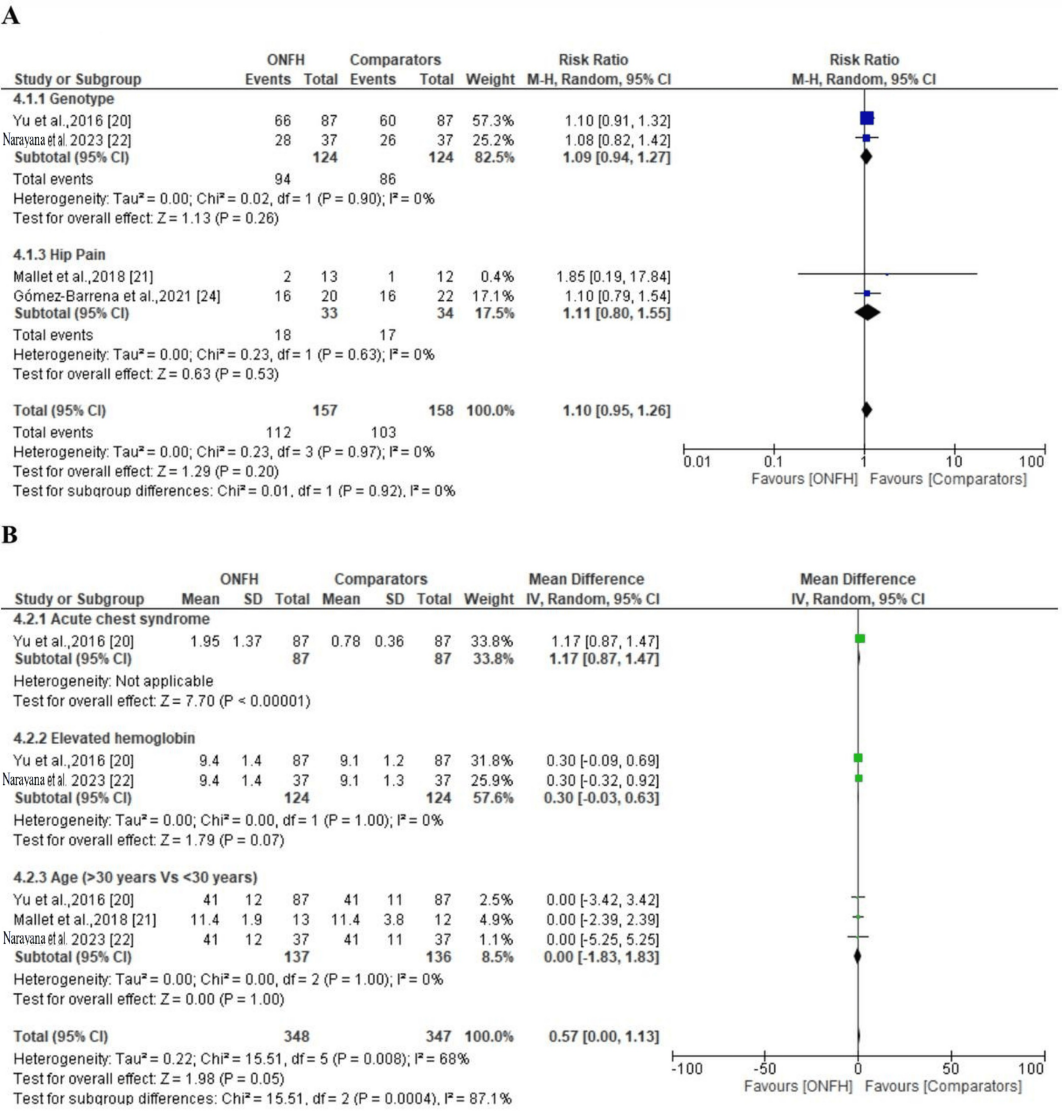
The common risk factors reported in the studies included in assessment of osteonecrosis (A) and the attributed risk factors (B). ONFH: osteonecrosis of the femoral head; M-H: Mantel-Haenszel method

The meta-analysis results indicate the heterogeneity for the test for the risk factors, such as genotype, elevated hemoglobin, hip pain, acute chest pain, and age, was statistically significant (p < 0.05), indicating high level of heterogeneity. The I^2^ for the five factors was 0%. On the other hand, the analysis of included studies revealed that all six assessed risk factors (genotype, hip pain, acute chest syndrome, elevated hemoglobin, and age) were identified as independent risk factors for the development of ONFH in individuals with sickle cell. The diamond in a meta-analysis forest plot symbolizes the overall effect estimate and its 95% confidence intervals. A skew towards the comparator group indicates that the assessed risk factor has a weaker association or lower impact on the ONFH group compared to the comparator group, suggesting its limited relevance in ONFH patients (Figures 4A, 4B) [17-19,21].

Discussion

This comprehensive review and meta-analysis identified low heterogeneity among studies assessing risk factors for ONFH in SCD including elevated hemoglobin levels, genotype, acute chest pain, age, and hip pain. All factors demonstrated consistent findings with an I² value of 0%. Similarly, a study by Mukisi-Mukaza et al. established a link between blood viscosity and ONFH, where elevated hemoglobin exacerbated femoral head microvascular obstruction, and leg ulcers further increased the risk of ONFH [27]. In a study by Yu et al., it is noted that acute complications in SCD patients increase the risk of ONFH, leading to increased hospitalizations and ER visits [20].

This study also found low heterogeneity, which was statistical significance in risk factors, such as hip pain, acute chest pain, and age, in relationship to femoral head osteonecrosis in SCD. This is in line with a study by Mallet et al., which found that interventions for ONFH in SCD patients, like hip replacements, resulted in good functional outcomes [21]. Further, Narayana et al.'s study highlighted symptomatic AVN as a risk factor for increased acute care utilization, emphasizing the need for early diagnosis and treatment [22].

The results of this study also revealed that increased hemoglobin levels, hip pain, acute chest syndrome, and genotype are the four main risk factors for osteonecrosis of the femoral head (ONFH) in sickle cell disease (SCD) patients. These risk factors are in line with earlier studies, such as the one conducted by Adesina et al., which discovered that those with more severe types of SCD and a history of acute chest syndrome were more likely to acquire ONFH, especially before the age of 30 years [1]. This could be explained by the fact that more severe types of SCD probably cause more microvascular damage, which can interfere with the femoral head's blood flow and raise the risk of ONFH. Furthermore, by worsening circulation and oxygen supply to the bones, acute chest syndrome, which includes pulmonary and vascular compromise, may make this process worse and contribute to the development of ONFH.

Further highlighting the complex nature of ONFH, Farook et al. found that 17.6% of patients who had surgery needed revision procedures, some as a result of complications such as prosthetic joint infections and acetabular osteolysis. According to these results, surgery may not always be a conclusive strategy for controlling ONFH in patients with sickle cell disease (SCD), especially if the underlying risk factors are not treated [23]. In terms of genotype, a number of studies have indicated that certain genetic variants in SCD may raise the risk of ONFH. Although the genotypes examined in this study were not specified, earlier studies have found some variants to be risk factors, such as those linked to hemoglobin S or elevated amounts of fetal hemoglobin. The severity of SCD and the likelihood of vascular injury in the femoral head may be influenced by these genetic variants, which highlights the necessity for individualized strategies in the prevention and management of ONFH in SCD patients.

Risk factors of ONFH had a smaller impact on the comparator group compared to the affected group. This finding is supported by the study by van Tuijn et al., who observed that over a seven-year period, organ damage in SCD patients increased, even though hospitalization rates for vaso-occlusive crises remained stable, with 62% of patients experiencing additional complications [28]. Furthermore, a study by Daltro et al. highlighted the potential benefits of concentrated bone marrow mononuclear cells (BMMCs), enriched with stem and endothelial progenitor cells, in promoting bone healing [29]. Similarly, Gómez-Barrena et al. reported favorable outcomes with advanced therapeutic products (ATMP), where 16 out of 20 patients showed significant improvement, although some required total hip replacement [24]. These findings emphasize the importance of early intervention for managing ONFH in SCD, even as innovative therapeutic approaches continue to show promise.

Moreover, a study by Tanabe et al. found that thrombosis, infarction, and necrosis in the bone's microcirculation could result from vaso-occlusive episodes (VOEs) in SCD, which is a significant factor in the development of ONFH. This emphasizes how vaso-occlusion affects bone health in individuals with SCD [25]. In contrast, Ilyas et al. found that surgery for ONFH resulted in a high survival rate of 98% over 10 years, along with notable improvements in hip scores, range of motion, and function. However, several problems were noted, including hip fractures, infections, aseptic shell failure, and 3.76% Brooker grade IV heterotopic ossification. These issues highlight the necessity of cautious management and monitoring even following surgery [30]. Based on this finding, it is commendable to say that there are several risk factors associated with ONFH in patients with SCD.

The strength of this study is its consistent and low heterogeneity on risk factors for osteonecrosis of the femoral head in sickle cell disease (SCD), which offers a strong basis for clinical practice and policy formation. It also emphasizes how crucial early intervention and cutting-edge treatment alternatives are. Nonetheless, the study was marred by a few limitations. First, it relied on existing literature, which may have inconsistencies in the quality and reporting of data. The limited availability of longitudinal studies on osteonecrosis of the femoral head (ONFH) in sickle cell disease (SCD) patients may restrict the ability to generalize findings. Secondly, although low statistical heterogeneity was observed (I² = 0%), methodological variations, including differences in sample sizes, diagnostic criteria for ONFH, and study populations, could affect the reliability of the pooled results. Future studies should adopt uniform criteria for diagnosing ONFH and reporting outcomes to enhance comparability and reliability across studies.

## Conclusions

This meta-analysis identified four significant risk factors of ONFH in SCD as follows: elevated hemoglobin levels, hip discomfort, acute chest pain, and genotype. The ONFH group demonstrated greater vulnerability to these factors compared to the comparator group. These findings underscore the need for proactive monitoring of key risk factors, including genotype and hemoglobin levels, and targeted interventions to address consistent indicators like hip pain and acute chest syndrome. In order to prevent problems and enhance patient care, the study highlights the significance of genotype, hemoglobin levels, and ONFH markers in clinical practice standards and public health policy. Further research should focus on refining treatment strategies and exploring additional risk factors to improve patient outcomes.
